# Account of Diseases in an Eastern District of London, from the 20th of December, 1801, to the 20th of January, 1802

**Published:** 1802-02

**Authors:** 


*Account of Diseases in an Eastern Diſtrict of London, from the* 20*th of December*, 1801, *to the* 20*th of January*, 1802.

| | | | |
|---|---|---|---|
| ACUTE DISEASES. | | Hemicrania - - - - - - | 2 |
| Typhus - - - - - - - | 3 | Paralyſis - - - - - - | 2 |
| Pneumonia - - - - - - | 5 | Vomitus - - - - - - - | 3 |
| Peripneumonia Notha - | 7 | Hæmorrhois - - - - - | 2 |
| Cynanche - - - - - - | 2 | Dyſpepſia - - - - - - | 5 |
| Rheumatiſmus Acutus - - | 3 | Enterodynia - - - - - | 4 |
| CHRONIC DISEASES. | | Dyſuria - - - - - - - - | 1 |
| Tuſſis - - - - - - - - | 25 | Chloroſis - - - - - - | 7 |
| Catarrhus - - - - - - | 5 | Amenorrhœa - - - - - | 8 |
| Dyſpnœa - - - - - - - | 12 | Lumbago - - - - - - | 3 |
| Tuſſis cum Dyſpnœa - - | 11 | Rheumatiſmus Chronicus | 25 |
| Aſthma - - - - - - - - | 2 | PUERPERAL DISEASES. | |
| Pleurodyne - - - - - - | 5 | Ephemera - - - - - - | 3 |
| Hæmatemeſis - - - - - | 1 | Menorrhagia Lochialis - | 4 |
| Phthiſis Pulmonalis - - | 4 | Dolores Poſt Partum - - | 3 |
| Hydrothorax - - - - - | 3 | INFANTILE DISEASES. | |
| Anaſarca - - - - - - | 4 | Diarrhœa - - - - - - | 6 |
| Palpitatio - - - - - - | 1 | Febris Meſenterica - - | 1 |
| Ophthalmia - - - - - - | 4 | Pertuſſis - - - - - - - | 5 |
| Cephalalgia - - - - - - | 3 | Tinea - - - - - - - - | 3 |

The ſtate of the weather during the laſt few weeks has, as it might be expected, been productive of thoſe diſeaſes which have their ſeat in the cavity of the thorax. Not only did the ſeverity of the cold occur at a very early period, but very quick changes have alſo taken place, during this period, in the ſtate of the atmoſphere. The alternations of freezing and thawing have been very frequent; a material change of temperature has been been felt within a few hours. Under all theſe circumſtances it is not ſurprizing that the diſeaſes which uſually prevail at this ſeaſon ſhould now make their appearance, nor that their ſymptoms ſhould be conſiderably aggravated. Accordingly, we find that catarrhal affections, coughs, peripneumonies, and other complaints of the cheſt, are very general. The ſeverity of the ſeaſon has proved fatal to a number of thoſe who were far advanced in life, and eſpecially to thoſe who have been for ſome time ſubject to affections of the cheſt at this ſeaſon of the year. In younger ſubjects, inflammations in this cavity have occurred in ſeveral inſtances; theſe have generally yielded to the uſe of leeches and bliſters, cathartics and antimonials.

It appears from the laſt yearly Bill of Mortality, that the number of deaths by the Small-pox was leſs by nearly one thouſand than in the preceding year. Whilſt we are aware that very little dependance is in general to be placed on a report taken in the manner in which that is taken from which the liſt of deaths is formed, yet we muſt acknowledge, with reſpect to the diſeaſe juſt mentioned, that it is not likely to be miſtaken, even by thoſe who are incompetent to give a proper report in other inſtances. May we not hope that the Vaccine Inoculation, as it is more generally adopted, will continue to diminiſh the influence, and finally to produce the total extirpation, of one of the moſt cruel and fatal diſeaſes to which the human frame is ſubject.

